# Oral Exposure to Epoxiconazole Disturbed the Gut Micro-Environment and Metabolic Profiling in Male Mice

**DOI:** 10.3390/metabo13040522

**Published:** 2023-04-05

**Authors:** You Weng, Ting Xu, Caihong Wang, Yuanxiang Jin

**Affiliations:** College of Biotechnology and Bioengineering, Zhejiang University of Technology, Hangzhou 310032, China

**Keywords:** mice, epoxiconazole, intestinal barrier, gut microbiota, metabolome

## Abstract

Epoxiconazole (EPX), a triazole fungicide, is widely used in agriculture to control pests and diseases. High residual and occupational exposure to EPX increases health risks, and evidence of potential harm to mammals remains to be added. In the present study, 6-week-old male mice were exposed to 10 and 50 mg/kg bw EPX for 28 days. The results showed that EPX significantly increased the liver weights. EPX also decreased the mucus secretion of the colon and altered intestinal barrier function in mice including a reduced expression of some genes (*Muc2*, *meprinβ*, *tjp1*). Moreover, EPX altered the composition and abundance of gut microbiota in the colon of mice. The alpha diversity indices (Shannon, Simpson) in the gut microbiota increased after exposure to EPX for 28 days. Interestingly, EPX increased the ratio of *Firmicutes* to *Bacteroides* and the abundance of other harmful bacteria including *Helicobacter* and *Alistipes*. Based on the untargeted metabolomic analysis, it was found that EPX altered the metabolic profiles of the liver in mice. KEGG analysis of differential metabolites revealed that EPX disrupted the pathway related to glycolipid metabolism, and the mRNA levels of related genes were also confirmed. In addition, the correlation analysis showed that the most altered harmful bacteria were associated with some significantly altered metabolites. The findings highlight that EPX exposure changed the micro-environment and lipid metabolism disturbance. These results also suggest that the potential toxicity of triazole fungicides to mammals cannot be ignored.

## 1. Introduction

For the past few years, large amounts of triazole fungicides have been approved for use in agriculture. With the increasing reports of fungicides, widespread safety concerns have been raised about the whereabouts of residues and the toxic effects of pesticide exposure [1,2]. Fungicides may enter the aquatic environment through spray drift, surface runoff, and rainfall [3]. Occupational exposure in agriculture increases the potential for toxic effects [4]. Epoxiconazole (1-[[3-(2-chlorophenyl)-2-(4-fluorophenyl) oxiran-2-yl] methyl]-1,2,4triazole, EPX), as a triazole fungicide, is one of the most widely used pesticides worldwide [5]. In killing fungi, it does so by directly inhibiting sterol 14α demethylase encoded by the CYP51A1 gene in humans. The blocking of sterol 14α demethylase results in a deficiency of ergosterol, which is essential for cell membranes in yeast and fungi [6,7,8]. It has been shown that fungicides have toxic effects on mammal organisms [9,10,11] and numerous studies have revealed that EPX produced brain, cardiac, liver, kidney, intestinal, and endocrine-disrupting toxicity [12,13,14,15,16,17]. There have been few studies on metabolic toxicity and intestinal barrier function impairment in mice.

Drugs entering the body by intragastric administration are common in vivo experiments of physiology, pathology, and toxicology [18]. The gastrointestinal tract is the main digestive organ, and its epithelial surface has a physical barrier that effectively absorbs nutrients from food [19]. The first probability is harmful substances in the gastrointestinal tract, which will have a toxic effect on the intestine [20]. Intestinal barrier function is influenced by endogenous and exogenous factors such as cytokines and chemicals [21]. A breakdown of the intestinal barrier can lead to tissue damage such as a disruption in mucus production [22,23]. The presence of rich flora in intestinal contents can ferment dietary fiber. The intestinal microbiome can also regulate intestinal motility and maintain the integrity of the intestinal epithelial biological barrier [24,25]. In addition, the intestinal microbiome influences gut and systemic health through its metabolites. Bacteria interact directly or indirectly with the host through secreted biologically active molecules, which may have protective or harmful effects on the intestinal barrier and various organs including the liver, kidney, and brain [26,27]. The liver interacts with the gut via the gut–liver axis.

With the development of mass spectrometry, the application of the metabolome in toxicological research has rapidly increased [28]. At environmentally relevant concentrations, exposed organisms respond more sensitively at the molecular level than at the organic level [29]. The implications of pesticides on biological metabolism should be addressed [30,31]. The metabolome based on LC-MS has been widely applied to explore changes in the liver metabolic profile [32,33,34]. Here, 6-week-old male C57BL/6 mice were orally administered 10 mg/kg bw, and 50 mg/kg bw EPX for 28 days. The growth phenotypes, biochemical indices, and intestinal barrier function were investigated. Gut microbiota is conducted to explore changes in the gut micro-environment. In addition, the untargeted metabolome of the liver can effectively reveal the difference in the metabolic profile of mice induced by EPX at the molecular level. This study aimed to reveal the effects of the pesticide EPX on the intestinal and hepatic toxicity in mice, mainly related to the effects of gut micro-environment and metabolic profile. The findings could provide evidence for the toxicological effects of the triazole fungicide EPX on the gut and liver in mammals, which can provide support for assessing the health risks of fungicides.

## 2. Materials and Methods

### 2.1. Chemical

EPX (CAS No.: 106325-08-0, purity ≥ 95%) and corn oil (CAS No.: 8001-30-7, medical-grade) were purchased from Aladdin Chemical Reagent Industry (Shanghai, China) and stored at 4 °C.

### 2.2. Animals and Study Design

The five-week-old male C57BL/6 mice (*n* = 24) were supplied by the China National Laboratory Animals Resource Center (Shanghai, China). The mice were randomly assigned to cages (a photoperiod of 12:12-h light/dark cycle, 22 ± 2 °C). Water and food were freely available throughout the feeding process. After a week of acclimatization, all mice were randomly placed into three treatment groups: Con, EPX-L (10 mg/kg bw), and the EPX-H group (50 mg/kg bw) (*n* = 8). The 100 μL corn oil with doses of 0, 10, 50 mg/kg bw were administered intragastrically daily, respectively, and the doses were selected based on the LD50 (>5000 mg/kg) of the rat. The duration of the gavage was 28 days, as a sub-chronic exposure test. Each mouse could drink pure water and consume commercial feed freely.

After 28 days of continuous gavage, all mice were sacrificed and dissected. The mice were fasted for 12 h, anesthetized with ether, then blood was taken from the eyeballs, and the neck was pulled to death. Serum was collected from the whole blood centrifugated (7000 rpm) at 4 °C for 10 min. The killed mice were dissected promptly, and the organs were weighed and immediately placed in liquid nitrogen. The serum and rapid-freezing organs were deposited at −80 °C for further testing. All mouse experiments were authorized by the Guiding Principles of Zhejiang University of Technology (20211117091).

### 2.3. Histological Analysis of the Liver and Colon

The liver and colon (*n* = 4) selected from the Con and EPX-H treatment group were randomly used for histological analysis. Briefly, the liver and colon were fixed in 4% paraformaldehyde overnight, embedded within paraffin wax, and cut into 5 µm-thick sections. The 5 µm-thick sections of livers were stained with hematoxylin and eosin (H&E), and the sections of the colon were stained with Alcian blue-periodic acid Schiff (AB-PAS). ImageJ2 was used for the quantitative analysis of AB-PAS staining.

### 2.4. Immunohistochemical and Immunofluorescence Analysis of the Colon

The pre-processing was the same as the previous steps to obtain 5 µm-thick sections of colons. For the immunohistochemical analysis, the rabbit anti-Muc2 (GB11344, Servicebio, Wuhan, China) primary antibody was applied at 1:500, then stained with FITC-conjugated (GB25303, Sevicebio) anti-rabbit secondary antibody. For the immunofluorescence analysis, the rabbit anti-claudin-1 (GB11032, Servicebio) and -ZO-1 (GB111402, Servicebio) primary antibodies were applied at 1:500, then stained with the HRP-conjugated (GB23303, Servicebio) anti-rabbit IgG secondary antibody. All images were captured with a fluorescence microscope (Nikon, Tokyo, Japan).

### 2.5. Biochemical Evaluation of the Serum and Hepatic Indices

The serum aspartate aminotransferase (AST) and alanine aminotransferase (ALT) were evaluated to assess liver function. The serum and hepatic triglyceride (TG), total cholesterol (TC), glucose (Glu), pyruvate (PYR), high-density lipoprotein cholesterol (HDL-C), and low-density lipoprotein cholesterol (LDL-C) were measured to evaluate the changed biochemical indices of the mice. The serum samples were directly used to detect the liver function and biochemical indicators. Liver samples were processed by adding PBS at 1:9 for grinding and centrifuging at ‘1000× *g* for 10 min. The supernatant was used to assess the biochemical indicators. The above tests followed the instructions in the commercial kits purchased from Nanjing Jiancheng Institute of Biotechnology (Nanjing, China). In addition, the protein concentration in the liver was detected with a Bradford Protein Assay Kit (Beyotime, Shanghai, China) for the relative quantification of each indicator.

### 2.6. RNA Extraction and Quantitative Real-Time PCR

The total RNA was extracted from the liver and colon of mice with the TRIZOL reagent. All extraction steps followed the instructions, and chloroform, isopropyl alcohol, and 75% alcohol were prepared. A Nano-300 (ALLSHENG, Hangzhou, China) detected the RNA concentration and purity. Furthermore, 1000 ng RNA was used for the cDNA synthesis using a reverse transcription kit. The next step was RT-qPCR, which was performed with the SYBR green system. All reagents were purchased from Vazyme (Nanjing, China), and all operations were conducted according to the instructions. Here, the specific primer sequences of the gene are listed in Table S1, and the synthesis was performed by Sangon Biotech (Shanghai, China). The expression level of β-actin was used to normalize the expression of specific genes. The PCR protocol followed the previous study [35]. The relative quantification of genes in all treatment groups was based on an earlier study [36].

### 2.7. 16S rRNA (V3-V4 Region) Sequencing and Data Analysis

The V3–V4 region of 16S rRNA sequencing was used to explore the intestinal microflora changes between the Con and EPX-H treatment groups. The total genomic DNA was extracted from the colon contents using a magnetic bead extraction method in our previous study [37]. Then, the DNA was amplified with specific primers of bacteria 16S rRNA (V3–V4, 338F: 5′-ACTCCTACGGGAGGCAGGAG-3′; 806R: 5′-GGACTACHVGGGTWTCTAAT-3′). The DNA amplification program was conducted according to a previous study [35]. Finally, the PCR products were purified with a Qiagen Gel Extraction Kit (Qiagen, DUS, Germany). The next step was sequencing library construction with the TruSeq^®^ DNA PCR-Free Sample Preparation Kit and adding index codes. The Qubit@ 2.0 Fluorometer (Thermo Scientific, Waltham, MA, USA) and Agilent Bioanalyzer 2100 system evaluated the library quality. The Illumina NovaSeq platform was used for high-throughput sequencing, and the 250 bp paired-end reads were obtained.

After data splitting and screening, sequence analysis was conducted with Uparse software (Uparse v7.0.1001) [38]. For the species annotation of 16S, the Silva Database was applied according to the Mothur algorithm. Alpha diversity analysis was calculated using the OIIME (Version 1.7.0) including PD_whole_tree, Shannon, Simpson, chao1, and ace. For the beta diversity, the OIIME (Version 1.9.1) was applied to calculate the unweighted unifrac. The linear discriminant analysis effect size (LEfSe) was used to detect the difference in taxonomies between the Con and EPX-H groups, which was convenient for finding the biomarker.

The microflora differences at the phylum level were determined by qPCR with DNA, and the primers are shown in Table S2. The protocol was performed as in the previous study [39]. The abundance of 16S was used to normalize the relative abundance of specific bacteria.

### 2.8. LC-MS-Based Metabolomics Analysis

The livers were used for metabolomics analysis based on LC-MS. A sample of 100 g of liver tissue was ground in tissue extract [75% (methyl alcohol: chloroform = 9:1): 25% H_2_O]. After sanding (50 Hz for 60 s, twice), the homogenate was treated with ultrasound for 30 min and placed on ice for 30 min before being centrifuged at 12,000 rpm for 10 min at 4 °C to achieve the supernatant for concentration and drying. The samples were redissolved using 50% acetonitrile solution with 2-chloro-L-phenylalanine solution (internal standard), which was used for LC-MS detection. The detection included chromatography conducted by the ultra-performance liquid system (Thermo Fisher Scientific, Waltham, MA, USA) and mass spectrometry was performed by the mass spectrometer detector (Thermo Fisher Scientific, Waltham, MA, USA). The above extraction, detection, and analysis work were all supported by Suzhou PANOMIX Biomedical Tech Co., Ltd. (Suzhou, China). Details are presented in the Supplementary Materials.

### 2.9. Statistical Analysis

Data were executed with GraphPad Prism 7.0 software and presented as the mean ± standard error of the mean (SEM). Data analysis between the Con and treatment groups was performed with one-way ANOVA followed by Dunnett’s post hoc test. Statistical significance was denoted with * (*p* ≤ 0.05) and ** (*p* ≤ 0.01).

## 3. Results

### 3.1. Oral Exposure to EPX Altered the Growth Phenotype of Mice

**Figure 1:**
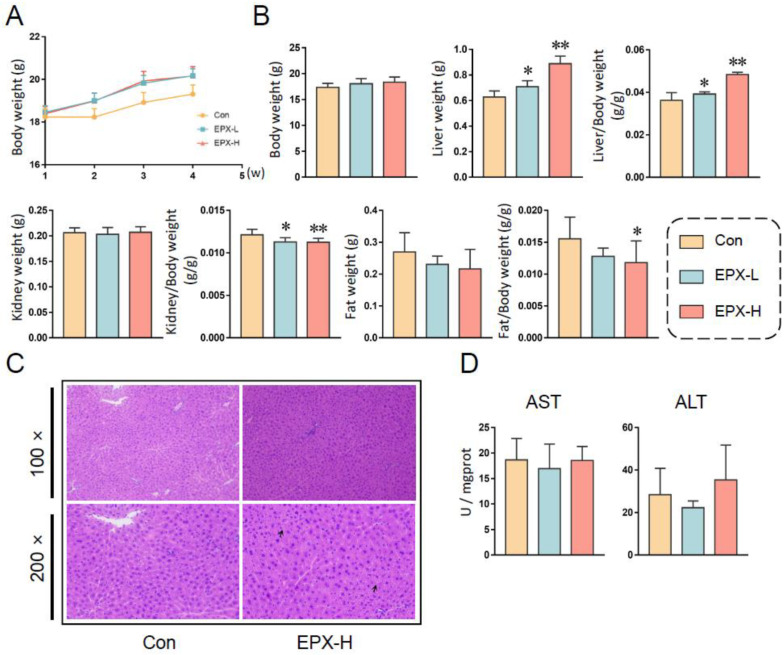
Effects of the oral exposure to EPX on the growth phenotype of mice. (**A**) The body weight of mice in each group weekly during exposure. (**B**) The body weight and organ weight of mice on the day of dissection. (**C**) The H&E staining of the liver. Black arrow: vacuolation. (**D**) Serum liver function indicators: AST activity, ALT activity. Values are shown as the means ± SEM (*n* = 8), and statistical significance: *p* < 0.05 *; *p* < 0.01 **.

After continuous EPX administration for 28 days, the body weights of mice showed an upward trend. However, there was no statistical significance (Figure 1A). Fasted for 12 h, the body weights of mice in different treatment groups did not change significantly (Figure 1B). However, the liver weight was elevated obviously (*p* < 0.05), with the increase in concentration as well as the ratio of liver to body weight (Figure 1B). Although the kidney weight did not change significantly, the ratio of the kidney to body weight decreased significantly (*p* < 0.05) in a concentration-dependent manner. Similarly, the fat weight did not vary, and the ratio of fat to body weight decreased (*p* = 0.035) in the EPX-H group.

In addition, there was no apparent pathological damage (vacuolization) to the liver of mice after being exposed to EPX (Figure 1C). The serum AST and ALT were also not altered significantly (Figure 1D).

### 3.2. EPX Altered the Biochemical Indices of Mice

For the serum biochemical indices (Table 1), the TC levels decreased significantly (*p* < 0.05) with the concentration dependence, and the TG and Glu levels did not change. The levels of PYR decreased (*p* = 0.03) in the EPX-L treatment group. Furthermore, HDL (*p* = 0.005) and LDL (*p* = 0.03) were lower in the high-concentration treatment group than in the other groups.

In addition, the hepatic biochemical indices were also detected (Table 1). The TC level was also decreased observably (*p* = 0.03) in the EPX-H treatment group. The TG, Glu, and PYR levels did not change after exposure to EPX. The HDL and LDL were not detected in the liver (not shown). Furthermore, we found that the NEFA level also decreased (*p* = 0.02) in the liver of mice exposed to EPX-H.

### 3.3. EPX Affected the Mucus Secretion and Tight Junctions in the Colon of Mice

**Figure 2:**
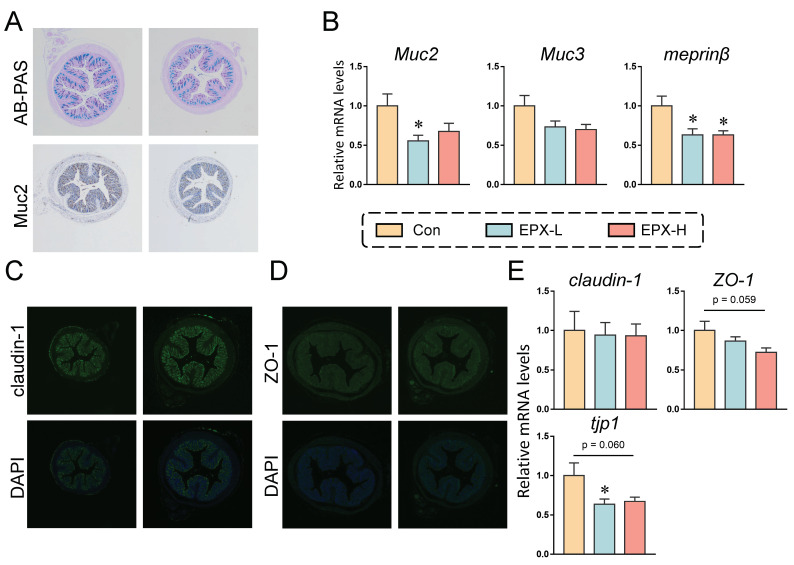
Effects of the oral exposure to EPX on the intestinal barrier function of mice. (**A**) AB-PAS staining and immunohistochemistry of Muc2 in the colon (*n* = 3). (**B**) The transcriptional levels of genes related to mucous secretion: *Muc2*, *Muc3*, and *meprinβ*. (**C**, **D**) Immunofluorescent staining of claudin-1 and ZO-1 (*n* = 3). (**E**) The transcriptional levels of genes related to tight junction: *claudin-1*, *ZO-1*, and *tjp1*. Values are shown as the means ± SEM (*n* = 8), and statistical significance: *p* < 0.05 *.

The mucus secretion of mice showed a numerical decrease in the EPX-H group compared to the Con group (Figure 2A and Figure S1A). Immunohistochemistry revealed that the expression of Muc2, a protein associated with the mucus secretion, also showed a downward trend, although there was no significant effect (Figure 2A and Figure S2B). We further detected the expression levels of genes involved in mucus secretion. As shown in Figure 2B, the expressions of the four genes detected were all decreased, among which, the expression level of *Muc2* in the colon of mice treated with EXP-L was significantly reduced (*p* = 0.026). Additionally, the transcriptional level of *meprinβ* was decreased in all EPX treatment groups (*p* = 0.016).

In addition, immunofluorescence revealed no significant changes in the claudin-1 and ZO-1 protein expression (Figure 2C,D). Similarly, the transcriptional levels of several genes associated with tight junctions were also examined. As shown in Figure 2E, the mRNA level of tjp1 was decreased in the EPX-L group (*p* = 0.040) and EPX-H (*p* = 0.060) group when compared to the Con group, and the mRNA level of ZO-1 was also decreased in the EPX-H group (*p* = 0.059).

### 3.4. EPX Regulated AMPs Expression and the Ionic Transport-Related Genes in the Colon of Mice

As shown in Figure S2A, the relative mRNA levels of genes related to *lyz*, *plazr4a*, and *ang4* were all increased significantly (*p* < 0.05) in the EPX-H treatment group. The other genes (*defα20*, *defα3*) did not change significantly.

Additionally, we analyzed the relative mRNA levels of genes involved in ionic transport in the colon of mice (Figure S2B). The results revealed that exposure to EPX had little effect on ion transport. Among the genes analyzed (*cftr*, *nkcc1*, *slc26a3*, *slc26a6*), only the mRNA level of *slc26a3* increased in a concentration-dependent manner (*p* < 0.05).

### 3.5. Oral Exposure to EPX Altered the Composition of Intestinal Microbiota in the Colon Contents

**Figure 3:**
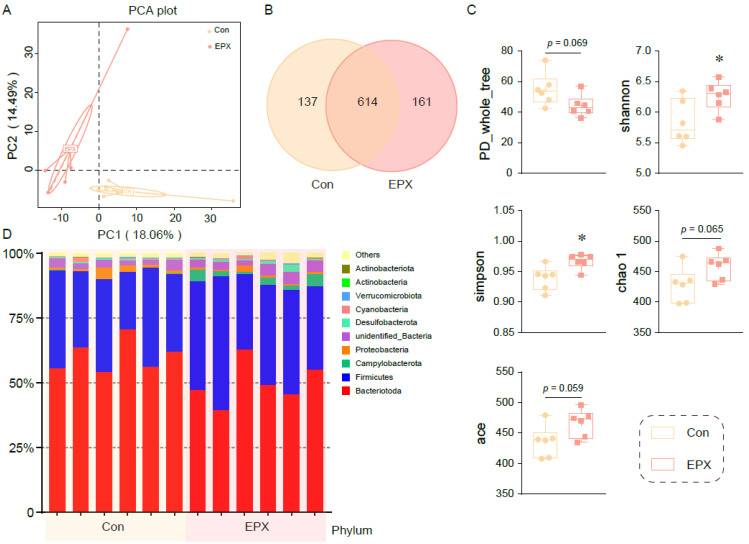
Effects of the oral exposure to EPX on the diversity of intestinal microbiota in the colon of mice (*n* = 6). (**A**) Principal coordinate analysis (PCoA) of OTUs. (**B**) Venn diagram of the OTUs numbers. (**C**) The relative abundance of the top—10 at the phylum level. (**D**) The indices of alpha diversity: PD_whole_tree, Shannon, Simpson, chao 1, ace. Values are shown as the means ± SEM (*n* = 6), and statistical significance: *p* < 0.05 *.

Principal coordinate analysis showed that the colonic microbiota in the EPX treatment group was different from that of the Con group (Figure 3A). After being exposed to EPX, the microbial composition changed, and the number of OTUs increased (Figure 3B). There was a difference in the affinities of species within the intestinal microbiota (PD_whole_tree: *p* = 0.069) of the two treatment groups. The Shannon and Simpson indices increased significantly (*p* < 0.05) in the EPX treatment group. Two other indices (chao1, ace) also increased, though not significantly. The above results show that EPX exposure increased the intestinal microbiota diversity in the colon content of mice. In addition, EPX disrupted the intestinal microbiota composition at the phylum level (Figure 3D). Specifically, the *Campylobacteria* and *Actinobacteria* levels increased in the EPX treatment group. Each of the other phyla had varying levels of change.

**Figure 4:**
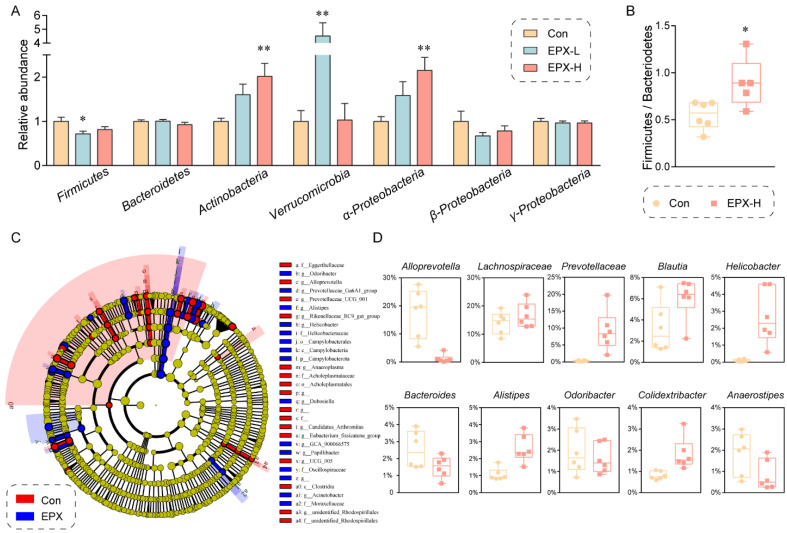
Effects of the oral exposure to EPX on the composition of intestinal microbiota in the colon of mice. (**A**) Relative abundance of the intestinal microbiota at the phylum level. Values are shown as the means ± SEM (*n* = 8), and statistical significance: *p* < 0.05 *; *p* < 0.01 **. (**B**) The ratio of Firmicutes to Bacteroides. (**C**) Cladogram generated from the LEfSe analysis (LDA score > 3). (**D**) The top-10 genera at the genus level.

Moreover, the difference in the intestinal microbiota in the colon contents between the EPX treatment group and the Con group was evaluated at the phylum level through RT-qPCR (Figure 4A). The relative abundance of *Actinobacteria*, *Verrucomicrobia*, and *α-Proteobacteria* was elevated significantly (*p* < 0.01) in the EPX-H treatment group. Although our RT-qPCR showed that only the relative abundance of *Firmicutes* declined (*p* = 0.03) in the EPX-L treatment group, 16S rRNA sequencing showed that the relative abundance ratio of *Firmicutes* to *Bacteroides* (*p* = 0.02) was significantly increased (Figure 4B). LEfSe analysis revealed that the relative abundance of *Campylobacteria* was increased significantly (Figure 4C). The relative abundance of some harmful bacteria such as *g_Alistipes*, *g_Helicobacter* was also increased. Specifically, other bacteria genera had been significantly altered (Figure 4D) including those upregulated (*Lachnospiraceae*, *Prevotellaceae*, *Blautia*, and *Colidextribacter*) and downregulated (*Alloprevotella*, *Bacteroides*, *Odoribacter*, and *Anaerostipes*).

### 3.6. Oral Exposure to EPX Disrupted the Metabolic Profile of the Liver

**Figure 5:**
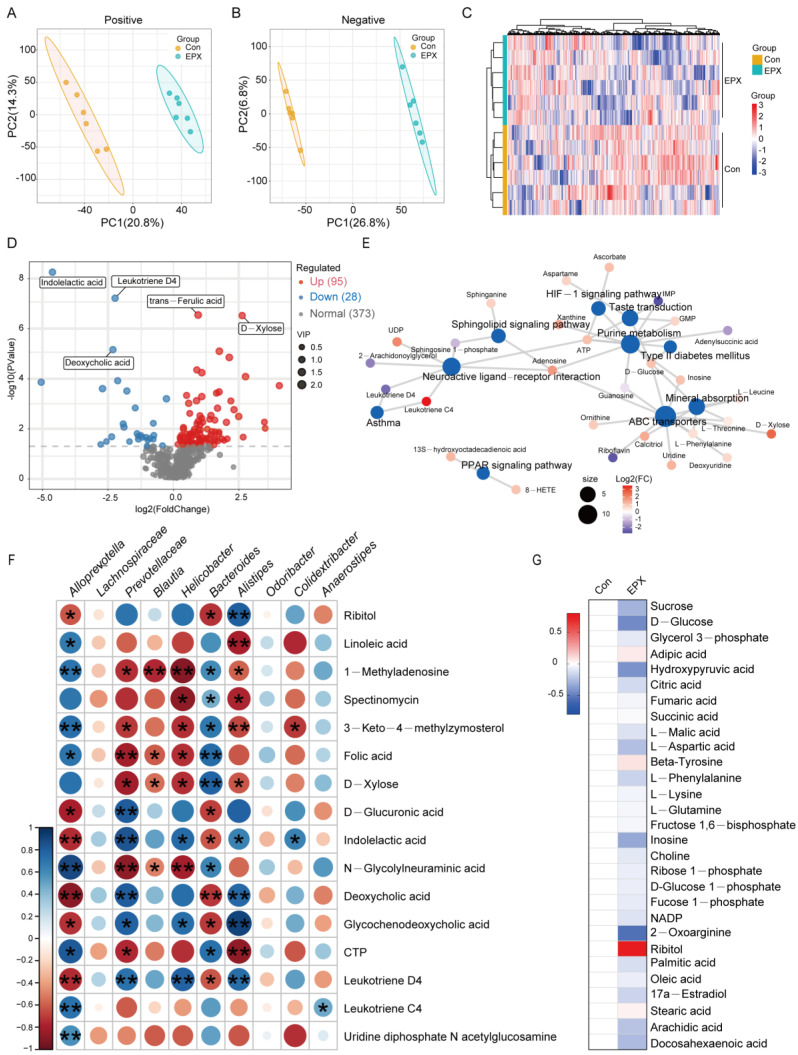
Effects of oral exposure to EPX on the metabolic profiles of the liver in mice. (**A**,**B**) PCA of metabolites in positive and negative ion mode. (**C**) Heatmap of DEMs. (**D**) Volcano map of DEMs. (**E**) Concept network of the KEGG pathway analysis for DEMs. (**F**) The correlation between the top genera conducted with the Spearman’s rank test. (**G**) Carbohydrate and lipid metabolites. Significant correlation: *p* < 0.05 *; *p* < 0.01 **.

Untargeted metabolomics based on LC-MS revealed differences in the metabolic profiles between the EPX treatment group and the Con group. In the negative and positive modes, the primary differential metabolites in the EPX treatment group showed better separation than that in the Con group (Figure 5A,B), and the metabolome data were reliable (Figure S3). As shown in the heatmap, MSMS secondary analysis suggested that samples from each treatment group in biological replications were clustered together by hierarchical clustering (Figure 5C). Among the identified metabolites, there was a total of 123 differential metabolites (DEMs) including 95 upregulated DEMs and 28 downregulated DEMs (Figure 5D). Some of the various metabolites included organic acids (indoleacetic acid, deoxycholic acid, trans-ferulic acid) and saccharides (D-xylose). In addition, the enrichment analysis of the KEGG pathway based on these DEMs showed that lipid metabolism-related metabolic pathways were the most affected including the PPAR signaling pathway and sphingolipid signaling pathway (Figure 5E). Interestingly, several bacteria with significant variations at the genus level were significantly correlated with differential metabolites such as *Alloprevotella*, *Prevotellaceae*, and *Bacteroides* (Figure 5F). In addition, through the screening of specific metabolites, we found changes in some major metabolites in glycometabolism (glycolysis) and lipid metabolism pathways such as citric acid, adipic acid, and ribitol (Figure 5G). 

### 3.7. Oral Exposure to EPX Affected the Transcription of Genes Related to Lipid Metabolism in the Liver

**Figure 6:**
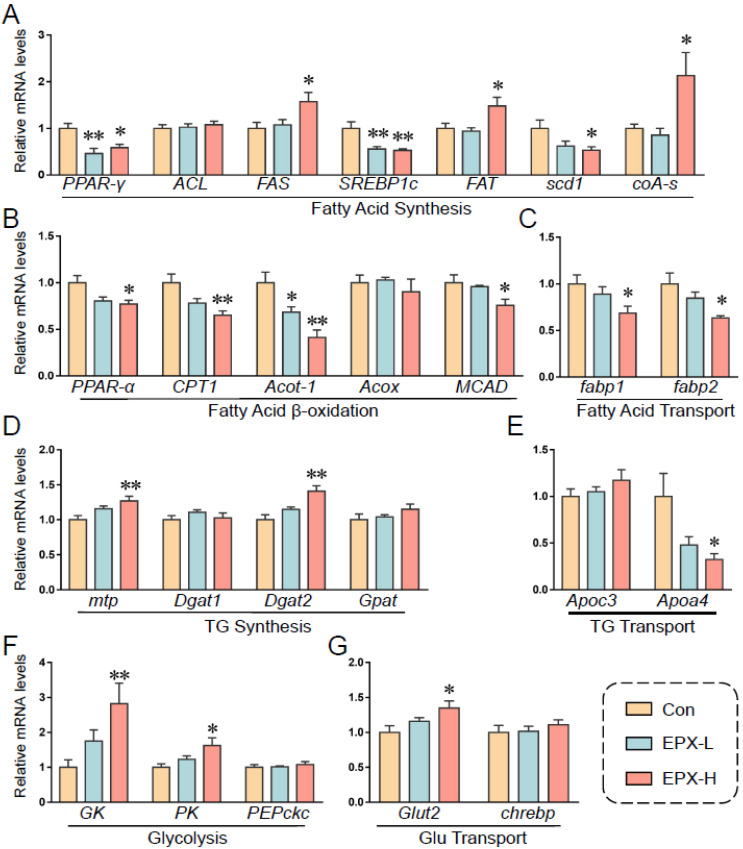
Effects of the oral exposure to EPX on the transcriptional levels of genes related to glycolipid metabolism. (**A**) Fatty acid synthesis. (**B**) Fatty acid β-oxidation. (**C**) Fatty acid transport. (**D**) TG synthesis. (**E**), TG transport. (**F**) Glycolysis. (**G**) Glu transport. Values are shown as the means ± SEM (*n* = 8), and statistical significance: *p* < 0.05 *; *p* < 0.01 **.

Based on previous results, we further detected the transcription levels of genes involved in lipid metabolism (Figure 6). EPX downregulated the expression of *PPAR-γ*, *SREBP1c*, and *scd1*, and upregulated the expression of *FAS* and *FAT*, which are involved in fatty acid synthesis (Figure 6A). Notably, the relative expression of *coA-s*, which synthesizes and oxidizes fatty acids and pyruvate, was markedly decreased. The expression of genes involved in lipid β-oxidation was also downregulated including *PPAR-α*, *CPT1*, *acot-1*, and *MCAD* (Figure 6C). The transcription levels of *fatp1* and *fatp2* were also decreased significantly in the EPX-H treatment group, which are involved in fatty acid transport (Figure 6D). Furthermore, the transcription levels of the TG synthesis-related genes were less affected, and the *DGAH* mRNA level was decreased in the EPX-H treatment group (Figure 6E). For the glycolysis and Glu transport, the relative mRNA levels of *GK*, *PK*, and *Glut2* were increased significantly in the EPX-H treatment group (Figure 6F,G).

## 4. Discussion

Health risk assessments for pesticides are always in progress. As one of the most widely used agricultural fungicides, triazoles account for over a quarter of the global sales of fungicides [40]. Their structures are similar to medical triazole drugs and have raised public health concerns [2,41]. Here, we assessed the potential toxicity of the triazole fungicide EPX in mammals. We found that the oral administration of EPX altered the growth phenotype and biochemical indices of the mice. EPX also induced enterotoxicity including impairment of the intestinal barrier function in the colon and intestinal microbiota dysbiosis. More importantly, untargeted metabolomics revealed changes in the liver metabolic profiles of the mice, affecting the lipid metabolic-related metabolic pathways.

The growth phenotype can first reflect the toxic effects of exogenous substances. EPX did not change the body weight of mice throughout the exposure period (Figure 1A). However, EPX significantly increased the liver weight and liver/body weight ratios and decreased kidney/body weight ratios in a concentration-dependent manner (Figure 1B). As previously reported, the liver weights and liver/body weight ratios increased significantly in the CD-1 mice fed with EPX at 50, 200, and 500 ppm [42]. EPX also increased the absolute and relative liver weight in the mice and rats [14,43]. This also supports that EPX does induce hepatotoxic effects. Although the serum AST and ALT levels also did not change in our study (Figure 1D) as well as in the existing findings in rat [43], the hepatotoxicity of triazole fungicides has previously been established [44,45,46,47]. After 28 days of EPX exposure, the AST and ALT levels were significantly increased, and liver function was disturbed [14]. This may be due to the significant individual differences in the mice in our study, and the error of the measured results was significant, failing to obtain the changes in these two levels. In addition, the serum and hepatic biochemical indices were also detected. The serum TC levels decreased with increasing EPX exposure concentration, and the hepatic TC level decreased significantly (Table 1). The same result was confirmed by the lower serum TC levels in the mice treated with 200 and 500 ppm EPX [42]. However, the biochemical indices of the parents would have the opposite trend of change [47]. The phenomenon also had the same conclusion in our previous experiments on the passage of maternal exposure [48]. The above results indicated that EPX can change the growth phenotype and physiological indices of the mice and may cause liver toxicity.

The gut is a vital tissue that can prevent harmful substances from entering the body’s circulation and acts as an intestinal barrier [49]. The intestinal barrier prevents foreign substances from entering the body through microbial recognition, mucus secretion, tight junction, AMP production, and ion transport [20]. Numerous pollutants can disrupt the intestinal barrier function and homeostasis of intestinal microbiota such as pesticides and microplastics [50,51]. The mucus layer is the physical line of defense of the gastrointestinal tract, and the Muc2 protein is the major structural component of the mucus [52,53]. We found that EPX reduced the colon mucus secretion in mice, and the expression of *Muc2* was obviously decreased in the EPX-L treatment group (Figure 2A,B). Furthermore, *meprinβ*, a gene related to mucus secretion, was significantly reduced in the EPX treatment group. An impaired tight junction is another major cause of barrier dysfunction, resulting in increased intestinal permeability. The Zo-1 anchors membrane proteins (claudin-1), the main component of tight junctions, on the actin cytoskeleton to maintain the integrity of interepithelial connections [54,55]. The mRNA levels of tight junction-related genes were decreased, and EPX had a more significant effect on the expression of *tjp1* and *zo-1* genes (Figure 2E). In addition, some genes associated with AMP expression and ion transport were also affected, mainly *lyz*, *plazr4a* and *slc26a3*. A recent study suggested that EPX alters the pathological structure of the colon of mice including the reduction in mucosal width and villus, and causes the infiltration of intestinal inflammatory cells [56]. The same triazole fungicide, difenoconazole, and prothioconazole disrupted the intestinal barrier function in mice [51,57].

The structure of the intestinal flora of the mice was similar to that of human flora. *Firmicutes* are essential in energy absorption, diabetes, and obesity development [58]. *Actinobacteria* can prevent systemic gastrointestinal diseases [59], while *proteobacteria* can prepare the gut for the rigorous colonization of anaerobic bacteria required for healthy intestinal function [60]. An existing study revealed that the relative abundance of *proteobacteria* and *actinobacteria* increased significantly in the intestinal flora of IgA nephropathy patients [61]. In the present study, we found that the abundance of *actinobacteria* and *α-Proteobacteria* was increased in the EPX-H treatment group, and the abundance of firmicutes was decreased in the EPX-L treatment group (Figure 4A). The ratio of *Bacteroides* to *Firmicutes* was increased (Figure 4B), which may contribute to metabolic syndromes such as obesity and diabetes [62]. In addition, *Verrucomicrobia* is a mucin-degrading bacterium that lives in the mucus layer of the large intestine and is involved in maintaining gut integrity [63]. However, abnormally proliferative *Verrucomicrobia* will survive by over-depleting mucin and may cause damage to the intestinal barrier. The abundance of *Verrumicrobia* was increased in the colon of mice exposed to EPX-H. At the genus level, the abundance of some harmful bacteria grew, and that of beneficial bacteria decreased. For example, *alist3ipes* is associated with obesity, and *helicobacter* can cause digestive tract lesions [64,65]. *Bacteroides*, as beneficial bacteria, play an important role in immune system regulation [66]. In contrast, *Bacteroides* at the phylum level were elevated in the gut of EPX-exposed rats [43]. However, the genus level of *Bacteroides* in rats exposed to EPX for 6 weeks also decreased compared to the control samples [13]. This slight difference in genus level is not sufficient to cause a change in the phylum level, which may lead to a difference in the results. This could be a result of a difference between species. In short, EPX disrupts the intestinal flora and may cause metabolic disorders.

Furthermore, we used untargeted metabolomics to explore changes in the mouse liver metabolic profiles. Metabolomics based on liquid chromatography-mass spectrometer (LC-MS) is widely used for the metabolic profiling of urine, serum samples, or animal tissue extracts [67]. The present study found that EPX resulted in apparent changes in the overall metabolite levels in mice (Figure 5A,B). The 1H-NMR-based metabolomics revealed that EPX altered the adult male zebrafish’s metabolic spectrum, mainly through increased amino acid levels [68,69]. According to the results of the KEGG enrichment of DEMs, the levels of some amino acids changed significantly including ornithine, L-leucine, and L-phenylalanine (Figure 5E). The same study also found that EPX altered genes related to energy metabolism in adult zebrafish including mitochondrial respiratory chain, ATP synthesis, and fatty acid β-oxidative [68]. Consistent with our research, EPX also affected the PPAR signaling pathway in the liver of the mice offspring [47]. From the perspective of the metabolic pathway, EPX altered metabolites in the pathway associated with energy metabolism in the liver of mice such as ATP, IMP, GMP, and UDP. Based on the correlation analysis of the bacteria with the greatest variation in phylum level and the greatest differences in metabolites, we found that some bacteria were closely related to metabolites such as *Alloprevotella*, *Prevotellaceae*, *Helicobacter*, *Bacteroides*, and *Alistipes* (Figure 5F). We believe that the effects of EPX on the abundance of these harmful bacteria are strongly related to the toxic effect on the liver. In addition, numerous toxicological studies have used transcriptome analysis to effectively detect changes in biomolecular indicators of model organisms, which can explain the mechanism of toxic events [70,71]. The transcriptome is also a good method to further explore the effects of pesticides on the gene level of mice.

Some metabolites in glycolipid metabolism changed significantly (Figure 5G). Again, the transcriptional levels of genes involved in glycolipid metabolism were also affected by EPX in mice (Figure 6). Specifically, with an altered PPAR signaling pathway, we found that the gene expression of *PPAR-γ, scd1*, *PPAR-α*, *MCAD CPT1*, *acot-1*, and *fabp1*, decreased significantly, which classified into fatty acid synthesis, fatty acid β-oxidation, and fatty acid transport. Reduced expression levels of CPT1, AOX, and MCAD in adult zebrafish exposed to EPX were also confirmed [68]. In addition, the metabolome revealed that D-glucose decreased as did glucose in the serum and liver (no significant). It may be that the expression of genes involved in glycolysis (*GK*, *PK*) and Glu transport (*Glut2*) increased significantly. Our previous experiments on larval zebrafish have also shown that EPX reduced glucose levels and increased the expression of *GK* and *HK1* but decreased the *PK* expression levels [72]. Transcriptome analysis also suggested that EPX may alter glycolipid metabolic pathways in mice such as cholesterol metabolism as well as the PPAR signaling pathway above-mentioned [47]. It is also our finding that EPX induced glycolipid metabolism in the liver of mice.

## 5. Conclusions

The toxicity of EPX to the liver and gut of male C57BL/6 mice exposed for 28 days was studied with 16S sequencing and untargeted metabolomics. EPX increased the liver weight and decreased the relative kidney weight. EPX also altered the intestinal barrier function and induced gut microbiota dysbiosis. Furthermore, EPX disrupted the metabolic profile of the liver in mice and induced glycolipid metabolism disorder. These results clarify the effect of EPX on the gut, intestinal microenvironment, and liver metabolism of the mice and provide a more theoretical basis for the risk assessment of EPX.

## Figures and Tables

**Table 1:** Effects of EPX exposure on the biochemical indicators of mice.

Biochemical Indicators	Con	EPX-L	EPX-H
Serum			
TC	2.267 ± 0.042	2.038 ± 0.038 *	1.914 ± 0.083 **
TG	0.133 ± 0.006	0.117 ± 0.015	0.150 ± 0.009
Glu	6.115 ± 0.357	5.782 ± 0.406	5.605 ± 0.215
PYR	0.402 ± 0.013	0.359 ± 0.007 *	0.439 ± 0.013
HDL	3.119 ± 0.152	2.651 ± 0.176	2.302 ± 0.181 **
LDL	0.774 ± 0.039	0.762 ± 0.062	0.533 ± 0.081 *
Liver			
TC	0.028 ± 0.007	0.030 ± 0.004	0.021 ± 0.005 *
TG	0.148 ± 0.051	0.151 ± 0.031	0.124 ± 0.027
Glu	0.163 ± 0.013	0.188 ± 0.033	0.137 ± 0.014
PYR	0.011 ± 0.005	0.012 ± 0.002	0.008 ± 0.002
NEFA	0.058 ± 0.016	0.055 ± 0.016	0.038 ± 0.006 *

Full name of biochemical indicators: TG, Triglyceride; TC, Total cholesterol; Glu, Glucose; PYR, Pyruvate; HDL, High-density lipoprotein cholesterol; LDL, Low-density lipoprotein cholesterol; NEFA, Non-esterified fatty acid. Values are shown as the means ± SEM (*n* = 8), and statistical significance: *p* < 0.05 *; *p* < 0.01 **.

## Data Availability

Not applicable.

## Supplementary Materials

The following supporting information can be downloaded at: https://www.mdpi.com/article/10.3390/metabo13040522/s1, Method: LC-MS-based metabolomics analysis [73,74,75]; Figure S1: The ratio of the mucus secretion area of colon; Figure S2: Effects of EPX exposure on the AMPs and ionic transport of colon in mice; Figure S3: PCA score chart of quality control samples; Table S1: The primer sequence pairs used in the RT-qPCR of specific genes; Table S2: The primer sequence pairs used in the RT-qPCR of bacteria at phylum level.

## Abbreviations

TG	Triglyceride
TC	Total cholesterol
Glu	Glucose
PYR	Pyruvate
HDL	High-density lipoprotein cholesterol
LDL	Low-density lipoprotein cholesterol
NEFA	Non-esterified fatty acid
