# Supplementary material for: Oral Exposure to Epoxiconazole Disturbed the Gut Micro-Environment and Metabolic Profiling in Male Mice
Source: Metabolites. 2023 Apr 5;13(4):522. doi: 10.3390/metabo13040522 (PMC10144212; doi:10.3390/metabo13040522)
Supplement: Supplementary file 1 [file metabolites-13-00522-s001.zip › metabolites-2300809-supplementary.pdf]

Oral exposure to epoxiconazole disturbed the gut micro-environment and metabolic profiling in male mice

Content

1. Method: LC-MS-based metabolomics analysis

2. Table S1. The primer sequence pairs used in the RT-qPCR of specific genes

3. Table S2. The primer sequence pairs used in the RT-qPCR of bacteria at phylum level

4. Fig. S1. The ratio of the mucus secretion area of colon

5. Fig. S2. Effects of EPX exposure on the AMPs and ionic transport of colon in mice

6. Fig. S3. PCA score chart of quality control samples

1. Method: LC-MS-based metabolomics analysis

The livers were used for metabolomics analysis based on LC-MS. A sample of 100 g of liv-er tissue was ground in tissue extract [75% (methyl alcohol: chloroform = 9:1): 25% H2O]. After sanding (50 Hz for 60s, twice), the homogenate was treated with ultrasound for 30 min and placed on ice for 30 min—centrifuge at 12000 rpm for 10 min at 4 °C to achieve the supernatant for concentration and drying. The samples were redissolved using 50% acetonitrile solution with 2-chloro-L-phenylalanine solution (internal standard), which was used for LC-MS detection. The detection included chromatography conducted by the ultra-performance liquid system (Thermo Fisher Scientific, USA) with ACQUITY UPLC® HSS T3 (Waters, Milford, MA, USA) and mass spectrometry performed by the Vanquish UHPLC System (Thermo Fisher Scientific, USA). Data were acquired in positive [The mo-bile phase consisted of acetonitrile (B2) and 0.1% ammonium formate water (A2); gradient: 0~1 min, 2% B2; 1~9 min, 2%~50% B2; 9~12 min, 50%~98%B2; 12~13.5 min, 98% B2; 13.5~14 min, 2%~98%B2; 14~20min, 2%B2] and negative mode [The mobile phase con-sisted of acetonitrile (B3) and 5 mM ammonium formate water (A3); gradient: 0~1 min, 2% B3; 1~9 min, 2%~50% B3; 9~12 min, 50%~98%B3; 12~13.5 min, 98% B3; 13.5~14 min, 2%~98%B3; 14~20min, 2%B3] [1]. The MSConvert tool in Proteowizard software package (v3.0.8789) was used to convert the raw data to mzXML format. The R XCMS software package was used for peak detection, peak filtration, and peak alignment to obtain a quantitative list of substances [ppm = 15, peakwidth = c (5, 30), mzwid = 0.015, mzdiff = 0.01, method = "centWave"]. Based on QC sample, the experimental data correction method of LOESS signal is described to eliminate the systematic error [2]. Materials with RSDS > 30% in QC samples were filtered out in data quality control. The metabolites were first identified according to the exact molecular weight, and then confirmed and annotated according to the MS/MS fragmentation model for HMDB (http://www.hmdb.ca), KEGG (http://www.genome.jp/kegg/), massbank (http://www.massbank.jp/), mzcloud (https://www.mzcloud.org), LipidMaps (http://www.lipidmaps.org), and self-established standard reference database. The

differential metabolites were found form the list of primary substances in the sample, and were screened according to preset P value (< 0.05) and VIP (> 1.0) in the statistical test [3].

1. Zelena E, Dunn W B, Broadhurst D, et al. Development of a Robust and Repeatable UPLC-MS Method for the Long-Term Metabolomic Study of Human Serum. *Anal. Chem.* **2009**, 81, 1357-1364.
2. Gagnebin Y, Tonoli D, Lescuyer P, et al. Metabolomic analysis of urine samples by UHPLC-QTOF-MS: Impact of normalization strategies. *Anal. Chima. Acta*. **2017**, 955:27-35.
3. Kieffer D A, Piccolo B D, Vaziri N D, et al. Resistant starch alters gut microbiome and metabolomic profiles concurrent with amelioration of chronic kidney disease in rats[J]. *Am. J. Physiol. Renal. Physiol*. **2016**, 310, F857-71.

2. Table S1. The primer sequence pairs used in the RT-qPCR of specific genes

Gene	Sequence of the primers (5'-3')
MCAD	F: 5'-GAGCCTGGGAACTCGGCTTGA-3'<br>R: 5'-GCCAAGGCCACCGCAACTTT-3'
PK	F: 5'-CAACAGGAAGGGTGTGAACTTG-3'<br>R: 5'-ACAAAGGAGGCAAAGATGATGT-3'
GK	F: 5'-GGCCACCAAGAAGGAAAAGGT-3'<br>R: 5'-CCTCTCCCACTTTGACCAGCA-3'
FAT	F: 5'-TTGCTGCCTTCTGAAATGTG-3'<br>R: 5'-GCAGAATCAAGGGAGAGCAC-3'
SREBP1c	F: 5'-TATGGAGGGCATGAAACCCGAAGT-3'<br>R: 5'-TTGACCTGGCTATCCTCAAAGGCT-3'
Fabp1	F: 5'-GTCAGCTGTGGAAAGGAAGC-3'<br>R: 5'-GTCTCCAGTTCGCACTCCTC-3'
Fabp2	F: 5'-TAAAGTAGCCCCAACCACGA-3'<br>R: 5'-TTCGCTGATGCACTGCCTATG-3'
PPAR-α	F: 5'-CCTCAGGGTACCACTACGGAGT-3'<br>R: 5'-GCCGAATAGTTCGCCGA -3'
scd1	F: 5'-TCCCTCCGGAAATGAACGAGAGAA-3'<br>R: 5'-AGTGCAGCAGGACCATGAGAATGA-3'
CPT1	F: 5'-CGCACGGAAGGAAAATGG-3'<br>R: 5'-TGTGCCCAATATTCCTGG-3'
PPAR-α	F: 5'-CCTCAGGGTACCACTACGGAGT-3'<br>R: 5'-GCCGAATAGTTCGCCGA -3'
scd1	F: 5'-TCCCTCCGGAAATGAACGAGAGAA-3'<br>R: 5'-AGTGCAGCAGGACCATGAGAATGA-3'
PPAR-γ	F: 5'-TTCGCTGATGCACTGCCTATG-3'<br>R: 5'-CGAAGTTGGTGGGCCAGAA-3'
FAS	F: 5'- GCAGCAAGTGTCCACCAACAA-3'<br>R: 5'-CTCATCGGAGCGCAGGATAGA -3'
ACL	F: 5'-AGGAAGTGCCACCTCCAACAGT-3'<br>R: 5'-CGCTCATCACAGATGCTGGTCA-3'
Dgat1	F: 5'-GACGGCTACTGGGATCTGA-3'<br>R: 5'-TCACCACACACCAATTCAGG-3'
Dgat2	F: 5'-CGCAGCGAAAACAAGAATAA-3'<br>R: 5'-GAAGATGTCTTGGAGGGCTG-3'
coA-s	F: 5'-TGTGGCACCGGATGTCTTT-3'<br>R: 5'-GACCAGATACCACGTTCCTTCAA-3'
Gpat	F: 5'-CAACACCATCCCCGACATC-3'<br>R: 5'-GTGACCT TCGATTATGCGATCA-3'
mtp	F: 5'-CACTCAGGCAATTCGAGACA-3'<br>R: 5'-TATCGCTTTCTGGCTGAGGT-3'
Acox	F: 5'-GCCCAACTGTGACTTCCATT-3'<br>R: 5'-GGCATGTAACCCGTAGCACT-3'

Apoc3	F: 5’-GTGTTGCAGATGTGCCTGTT-3’<br>R: 5’-GGAGGGGTGAAGACATGAGA-3’
Apoa4	F: 5’-CGCAGCGAAAACAAGAATAA-3’<br>R: 5’-GAAGATGTCTTGGAGGGCTG-3’
Glut2	F: 5’-ACCCTGTTCCTAACCGGG-3’<br>R: 5’-TGAACCAAGGGATTGGACC-3’
PEPckc	F:5′-ATCACGCATCGCTAAAGAGG-3′<br>R:5′-CCGCTGCGAAATACTTCTTC-3
Muc2	F: 5’-TACGCTCTCCACCAGTTCCT-3’<br>R: 5’-CAGCTCTCGATGTGTGTGTAGGT-3’
Muc3	F: 5’-TGGTCAACTGCGAGAATGGA-3’<br>R: 5’-TACGCTCTCCACCAGTTCCT-3’
meprinβ	F: 5’-CAGGCAAGGAACACAACTTC-3’<br>R: 5’-TCTGTCCCGTTCTGGAAAG-3’
claudin-1	F: 5’-GATGTGGATGGCTGTCATTG-3’<br>R: 5’-CCTGGCCAAATTCATACCT-3’
ZO-1	F: 5’-TGGTCAACTGCGAGAATGGA-3’<br>R: 5’-TACGCTCTCCACCAGTTCCT-3’
tjp1	F: 5’-ACCCGAAACTGATGCTGTGGATAG-3’<br>R: 5’-AAATGGCCGGGCAGAACTTGTGTA-3’
cftr	F: 5’-AAGGCGGCCTATATGAGGTT-3’<br>R: 5’-AGGACGATTCCGTTGATGAC-3’
Nkcc1	F: 5’-CAAGGGTTTCTTTGGCTAT-3’<br>R: 5’-TCACCTGAGATATTTGCTCC-3’
slc26a3	F: 5’-GTCTACTGAACTTCGGGGTGAT-3’<br>R: 5’-GTAAAATCGTTCTGAGGCCCC-3’
slc26a6	F: 5’-CCAAACATAGGAGGCAATCC-3’<br>R: 5’-GGTATCCTGTGCGTGAATGGCTC-3’
lyz	F: 5’-GAGACCGAAGCACCGACTATG-3’<br>R: 5’-CGGTTTTGACATTGTGTTCGC-3’
pla2γ4a	F: 5’-GGCCTTTGGCTCAATACAGGTC-3’<br>R: 5’-ACAGTGGCATCCATAGAAGGCA-3’
Ang4	F: 5’-TGGCCAGCTTTGGAATCACTG-3’<br>R: 5’-GCTTGGCATCATAGTGCTGACG-3’
defα20	F: 5’-GTCCAGGCTGATCCTATCCA-3’<br>R: 5’-GATTTCTGCAGGTCCAAAA-3’
defα3	F: 5’-TCCTCCTCTCTGCCCTCGT-3’<br>R: 5’-GACCCTTTCTGCAGGTCCC-3’
GAPDH	F: 5’-GTGGCAAAGTGGAGATTGTTG-3’<br>R: 5’-AGTCTTCTGGGTGGCAGTGAT-3’

3. Table S2. The primer sequence pairs used in the RT-qPCR of bacteria at phylum level

Gene	Sequence of the primers (5’-3’)
Firmicutes	F: 5’-GGAGYATGTGGTTTAATTCGAAGCA-3’
	R: 5’-AGCTGACGACAACCATGCAC-3’
Bacteroidetes	F: 5’-GGARCATGTGGTTTAATTCGATGAT-3’
	R: 5’-AGCTGACGACAACCATGCAG-3’
Actinobacteria	F: 5’-CGCGGCCTATCAGCTTGTTG-3’
	R: 5’-ATTACCGCGGCTGCTGG-3’
α-Proteobacteria	F: 5’-ACTCCTACGGGAGGCAGCAG-3’
	R: 5’-TCTACGRATTTCACCYCTAC-3’
β-Proteobacteria	F: 5’-CCGCACAGTTGGCGAGATGA-3’
	R: 5’-CGACAGTTATGACGCCCTCC-3’
γ-Proteobacteria	F: 5’-GAGTTTGATCATGGCTCA-3’
	R: 5’-GTATTACCGCGGCTGCTG-3’
Verrucomicrobia	F: 5’- GAATTCTCGGTGTAGCA-3’
	R: 5’-GGCATTGTAGTACGTGTGCA-3’
16 S	F: 5’-ACTCCTACGGGAGGCAGCAG-3’
	R: 5’-ATTACCGCGGCTGCTGG-3’

4.

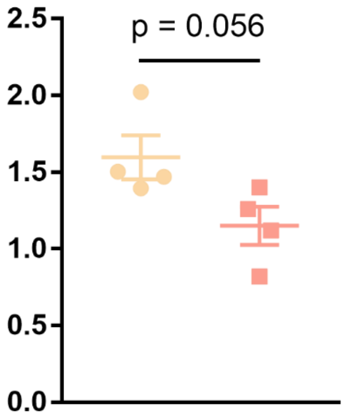

Figure S1. The ratio of the mucus secretion area of colon

5.

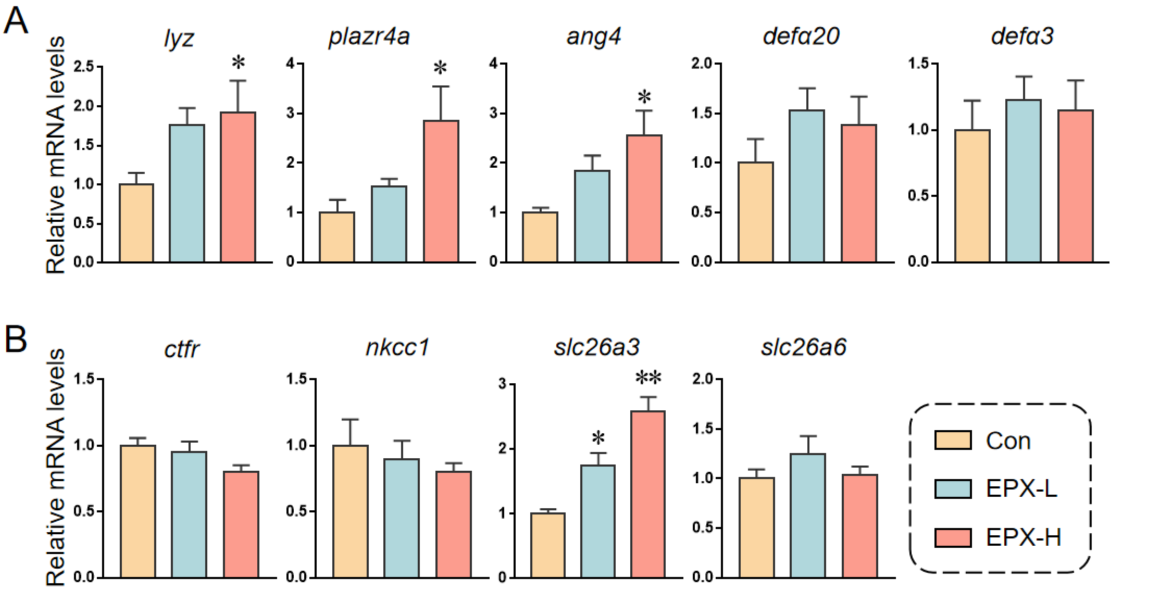

Figure S2. Effects of EPX exposure on the AMPs and ionic transport of colon in mice. A, the transcription levels of genes related to AMPs. B, the transcription levels of genes related to ionic transport. Values are shown as the means ± SEM (n = 8), and statistical significance: $p < 0.05$*; $p < 0.01$**.

6.

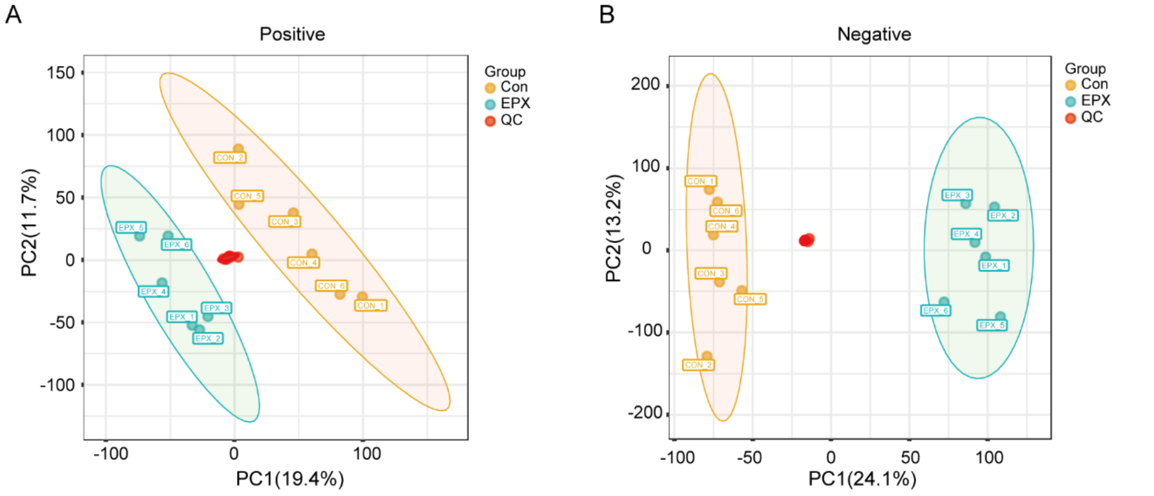

Figure S3. PCA score chart of quality control samples. A, positive mode; B, negative mode. QC, quality control.
